# A multi-channel photometric detector for multi-component analysis in flow injection analysis

**DOI:** 10.1155/S1463924694000088

**Published:** 1994

**Authors:** Aimin Tan, Jialin Huang, Liudi Geng, Jinhua Xu, Xinna Zhao

**Affiliations:** Center for Process Analytzcal Chemistry Department of Chemistry Central South Universily of Technology Hunan Changsha 410083 China

## Abstract

The detector, a multi-channel photometric detector, described in this paper was developed using multi-wavelength LEDs (light emitting diode) and phototransistors for absorbance measurement controlled by an Intel 8031 8-bit single chip microcomputer. Up to four flow cells can be attached to the detector. The LEDs and phototransistors are both inexpensive, and reliable. The results given by the detector for simultaneous determination of trace amounts of cobalt and cadmium in zinc sulphate electrolyte are reported. Because of the newly developed detector, this approach employs much less hardware apparatus than by employing conventional photometric detectors.


Journal of Automatic Chemistry, Vol. 16, No. 2 (March-April 1994), pp. 71-73

### A multi-channel photometric detector for multi-component analysis in flow injection analysis

**Aimin Tan, Jialin Huang, Liudi Geng, Jinhua Xu and Xinna Zhao**

*Center for Process Analytical Chemistry, Department of Chemistry, Central South University of Technology, Changsha, Hunan 410083, China*

*The detector, a multi-channel photometric detector, described in this paper was developed using multi-wavelength LEDs (light emitting diode) and phototransistors for absorbance measurement controlled by an Intel 8031 8-bit single chip microcomputer. Up to four flow cells can be attached to the detector. The LEDs and phototransistors are both inexpensive, and reliable. The results given by the detector for simultaneous determination of trace amounts of cobalt and cadmium in zinc sulphate electrolyte are reported. Because of the newly developed detector, this approach employs much less hardware apparatus than by employing conventional photometric detectors.*

### Introduction

The spectrophotometer is one of the most widely employed detectors in flow injection analysis (FIA). Traditionally, spectrophotometric detection is operated at only one wavelength at a time and multi-component analysis by FIA is achieved by exploiting the versatility of FIA's manifold [1]. Simultaneous determination of nitrate and nitrite, for instance, involves splitting the injected sample into two streams, one of which is equipped with a zinc reductor. The streams are then transported to the same detector one after another, recording two peaks. This approach is dependent on the manifold design, and will not work in many cases.

A recent development is the photodiode array (PDA) based detector, with which a sample's spectrum can be obtained very quickly; when used with FIA it simplifies multi-component analysis [2, 3]. However, the PDA-based detector is usually much more expensive than the conventional spectrophotometer; and where the colour-metric systems for each component are different, the PDA based detector does not work.

There are increasing numbers of examples of multi-component analysis in FIA being achieved with several detection points located in series or in parallel [4]. As the photometric detectors used for these are single-channel (i.e. they can equip or monitor only one flow cell at a time), several individual detectors need to be used together in order to accomplish mult-component analysis [5], which makes the instrument set-up not only more complicated and expensive, but also less reliable. So a single detector which could simultaneously monitor more than one flow cell at multi-wavelength for multi-component analysis would be of great advantage. Such a detector is described in this paper (background is given in Huang *et al.* [6]).

### Experimental

### *The photometric detector*

A diagram of the photometric detector is given in figure 1. Two flow cells are employed in the detector, each of which is equipped with a dual-wavelength LEDs and a phototransistor as light source and photodetector. All of the LEDs have their own driving circuits, which are connected to a D/A converter through an electronic multiplexer: CD4051. CD4051 has eight switches, so the total number of LEDs should be less than eight. If each flow cell is monitored at two different wavelengths, up to four flow cells can be connected to the photometer. The phototransistors are all connected to one amplifier. The LEDs are switched on and off sequentially, under microcomputer control, giving off different narrow-band light beams for absorbance measurement. After being absorbed by the solutions in the flow cells, the light beams fall on the corresponding phototransistors one by one. The current generated is then amplified by the amplifier, and digitized by a 12-bit A/D converter. Finally, the absorbance for each flow cell at each wavelength are calculated by the single-chip microcomputer. As all the procedures are carried out very quickly (in milliseconds), the flow cells can be simultaneously (or almost simultaneously) monitored. Because both the other associate

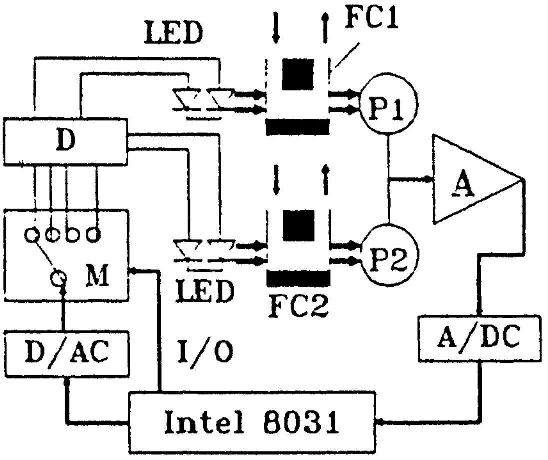

*Figure 1. The multi-channel photometric detector: M = analogue multiplexer (CD4051); D = driving circuits; LED = dual-wavelength LEDs; FC1 and FC2 = Hellma flow cells with 10 mm of optical length and 1·5 mm of diameter; P1 and P2 = phototransistors (3DU80B); A = amplifier (ICL7650); D/AC = digital to analogue converter (DAC0832); A/DC = analogue to digital converter (AD574); I/O = control signal for the CD4051; Intel 8031 = single-chip microcomputer based system.*

0142-0453/94 $10.00 © 1994 Taylor & Francis Ltd

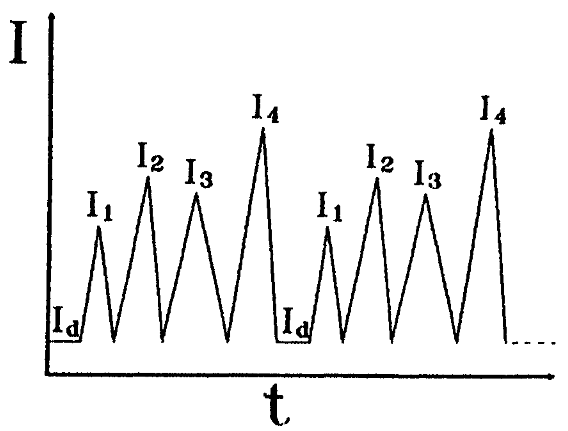

*Figure 2. Signal detected versus time. $I_d$ = dark current; I1–I4 = currents detected when different LEDs are switched on.*

electronics, such as A/D converter, D/A converter, amplifier, and the computer system, are shared, and the LEDs together with the phototransistors are small, cheap, and reliable, the detector is both simple and reliable.

Since light intensities from the different LEDs may be different, and because the sensitivity of the photodetector towards light with different wavelengths may be different, for each wavelength, the 100% transmittance must be calibrated separately. This is achieved by sending different digital numbers to the D/A converter to generate corresponding voltage or/and by running each LED for different durations.

While in operation, the LEDs are switched on and off one by one; an example of the resulting signal is given in figure 2.

Absorbance is calculated according to the following equation:

$$A = \log [(I_0 - I_d)/(I - I_d)] \quad (1)$$

Where $A$ is the absorbance for each wavelength in a given flow cell; $I_d$ is the dark current; and $I_0$ and $I$ are the digital signals (proportional to the light intensity) observed while the blank solution and the sample solution are captured in the flow cells, respectively.

### *The flow injection system*

An eight-channel peristaltic pump and eight-port rotary injection valve (Zhaofa Automatic Analysis Institute, Shengyang, China) are employed as the flow injection system.

### *Reagents and solutions*

1. $1{\cdot}6 \times 10^{-5}$ g/ml bromothymol blue + 0·01 mol/l Borax.

2. Cobalt stock solution, 100 μg/ml.

3. Cadmium stock solution, 100 μg/ml.

R1: 3% NaAc.

R2: 0·01% 5-Cl-PADAB (containing 10% of ethanol).

R3: 4% $H_2SO_4$

R4: 10% KI + 1% ascorbic acid + 0·3 mol/l $Na_2SO_4$ + 0·3% arabic gum (chemical grade) + 2% thiourea + 0·1 mol/l HAc-NaAc (pH = 5·0).

R5: 0·008% ethyl violet.

R6: 0·80 mol/l HCl.

All the reagents employed, except otherwise stated, were of analytical grade.

### Results and discussion

### *Absorbance measurement and precision*

The absorbances measured for a series of bromothymol blue solutions by using a red/green dual wavelength LEDs with the emission maxima at 630 nm are summarized in equations 2 and 3 [6]

$$A = 0{\cdot}0194 + 4{\cdot}909 \times 10^4 C \text{ (for 630 nm)} \quad (2)$$

$$A = 0{\cdot}0016 + 5{\cdot}474 \times 10^4 C \text{ (for 565 nm)} \quad (3)$$

Where $A$ is the absorbance and $C$ is the concentration of bromothymol blue in g/ml. The regression coefficients of equations 2 and 3 are 0·9994 and 0·9999, respectively. In order to demonstrate the precision, a blank and a dye solution of absorbance 0·794 at 630 nm and 0·875 at 565 nm were measured 11 times. For the blank the standard deviation of absorbance at 630 nm and 565 nm was 0·0009 and 0·0007, respectively; for the dye solution, the standard deviation of absorbance was 0·0020 at 630 nm and 0·0021 at 565 nm, with a resulting RSD of 0·25% and 0·24% for red and green colours, respectively, which can meet the precision demand in FIA (1%).

### *Coupled with FIA for simultaneously determining trace cobalt and cadmium*

In zinc hydrometallurgical plants, cobalt and cadmium content in purified zinc sulphate solutions directly affects the quality of cathode deposit and need to be controlled below a certain limit (0·001 g/l) before electrolysis. Recently, some methods based on FIA have been proposed for at-line or on-line analysis of these two elements [7, 8, 9]. Because the traditional photometer for FIA can monitor only one flow cell at a time, and the colourmetric systems for cobalt and cadmium are different, two separate detectors are needed to achieve the simultaneous determination of the two elements, resulting the instrument set-up more complicated and less reliable. The new photometer described in this paper requires substantially less instrument hardware for simultaneously determining cobalt and cadmium: only one peristaltic pump and one injection valve, together with the photometer are necessary (figure 3).

First, the two separate sampling loops on the valve are simultaneously injected, merging downstream with the corresponding reagents in two separate manifolds, and finally flowing to two different flow cells (one for cobalt, the other for cadmium). For each flow cell, a dual wavelength LEDs with the emission maxima at 630 nm and 565 nm is attached. The light beam with 565 nm of wavelength is employed as sample beam, and the light beam with 630 nm of wavelength is used as the reference beam to compensate the possible background absorption (for example, the sample's turbidity). The optical length of the two flow cells are all 10 mm. Because the spectral

*Table 1. The results of sample analysis (μg/ml) from a zinc hydrometallurgical plant.*

| Sample No. | Recommended value | | Determined value | |
|---|---|---|---|---|
| | Co | Cd | Co | Cd |
| 1 | 0·65 | 0·31 | 0·68, 0·67, 0·64, 0·65, 0·61 | 0·32, 0·30, 0·33, 0·34. 0·29 |
| 2 | 0·53 | 0·52 | 0·56, 0·55, 0·52, 0·50, 0·54 | 0·48, 0·50, 0·53, 0·55, 0·51 · |
| 3 | 0·47 | 0·40 | 0·52, 0·49, 0·45, 0·45, 0·44 | 0·36, 0·38, 0·41. 0·39, 0·43 |
| 4 | 0·13 | 0·72 | 0·18, 0·15, 0·15, 0·14, 0·13 | 0·68, 0·67, 0·70, 0·75, 0·71 |

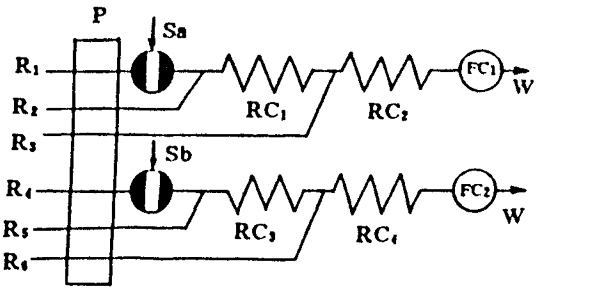

*Figure 3. FIA manifold for the determination of cobalt and cadmium. P = peristaltic pump; Sa and Sb = loaded samples of 40 μl volume; $RC_1$–$RC_4$ = reaction coils—diameter of 0·5 mm, and lengths 180 cm, 70 cm, 200 cm, and 80 cm, respectively; $FC_1$ and $FC_2$ = flow cells; W = waste; R1–R6 = reagents.*

intensity at 565 nm is smaller than that at 630 nm, the lighting time for 565 nm LED is 0·1 s, while the lighting time for 630 nm LED is 0·05 s. Therefore, the total time for each measuring cycle, obtaining the absorbances of the two flow cells at two wavelengths, is less than 0·35 s. Sampling frequency is three times/s, which can reach the speed demand for recording the FIA's response curve in this method, whose base to base time is about 15 s.

After the sample is injected, the detector will calibrate 100% transmittance and compensate the dark current first. Then, it begins to monitor the absorbance, and two peaks are recorded. The peak height is employed as the analytical readout.

Samples from a zinc hydrometallurgical plant have been analysed using the method described above—the results of this, together with results from traditional methods based on spectrophotometric detection of cobalt with nitroso R salt [10] and polarographic detection of cadmium [10], are listed in Table 1. The results show that the accuracy and precision of the new detector easily meet the requirements of plant analysis.

### References

1. Gine, M. F., Bergamin, F. E., Zagatto, A. G. and Reis, B. F., *Analytica Chimica Acta*, **114** (1980), 191.
2. Blanco, M., Coello, J., Gene, J., Iturriaga, H. and Maspoch, S., *Analytica Chimica Acta*, **224** (1989), 23.
3. Lindberg, W., Clark, G. D., Hanna, C. P., Whitman, D. A., Christian, G. D. and Ruzicka, J., *Analytical Chemistry*, **62** (1990), 849.
4. Ruzicka, J. and Hansen, E. H., *Flow Injection Analysis* (2nd edn), Eds Winefordner, J. D. and Kolthoff, I. M. (John Wiley & Sons, 1988), 222.
5. Chen, D., Cai, R. and Zeng, Y., *Analytica Chimica Acta*, **222** (1989), 205.
6. Huang, J. L., Liu, H. H., Tan, A. M., Xu, J. H. and Zhao, X. N., *Talanta*, **39** (1992), 589.
7. Li, W. C. and Fu, B., *Fenxi Shiyanshi* (in Chinese), **3** (1990), 5.
8. Li, W. C. and Fu, B., *Hengliang Fenxi* (in Chinese), **4** (1989), 55.
9. Wang, Z. H. *et al.*, *Fenxi Shiyanshi* (in Chinese), **7** (12) (1988), 35.
10. Zhuzhou Smeltery, *Metallurgical Analysis of Copper, Lead, and Zinc* (People's Press of Hunan, 1973), 146, 150.

